# Stratifying septic patients using lactate: severe sepsis and cryptic, vasoplegic and dysoxic shock profile

**DOI:** 10.1186/cc12937

**Published:** 2013-11-05

**Authors:** Otavio T Ranzani, Mariana B Monteiro, Elaine M Ferreira, Fernando Leibel, Sergio Ricardo Santos, Flavia R Machado, Danilo T Noritomi

**Affiliations:** 1 Hospital Paulistano, São Paulo, Brazil; 2 Disciplina de Emergências Clínicas, Hospital das Clínicas, Universidade de São Paulo, Brazil; 3 Latin America Sepsis Institute, São Paulo, Brazil; 4 Disciplina de Anestesiologia, Escola Paulista de Medicina, Universidade Federal de São Paulo, Brazil

## Background

The current consensus definition of severe sepsis and septic shock includes a heterogeneous profile of patients under the same definition. Although the prognostic value of hyperlactatemia in sepsis is well established, hyperlactatemia can be found both in severe sepsis and septic shock patients. We sought to compare features and outcomes of septic patients stratified by two factors: the presence of hyperlactemia and persistent hypotension.

## Materials and methods

This was a secondary analysis of a multicenter observational study from 10 private hospitals in Brazil (Rede Amil-SP) aiming to evaluate the impact of a multifaceted program to implement the Surviving Sepsis Campaign bundles. We retrieved 1,948 septic patients with an initial lactate level collected within the first 6 hours of diagnosis. Based on previous literature, we stratified them into four groups according to the presence of hypoperfusion (lactate >4 mmol/l) and/or persistent hypotension despite adequate fluids: 1, severe sepsis (without both criteria); 2, cryptic shock (hypoperfusion without persistent hypotension) [1]; 3, vasoplegic shock (persistent hypotension without hypoperfusion); and 4, dysoxic shock (with both criteria) [2].

## Results

Severe sepsis was found in 1,018 (52%), cryptic shock in 162 (8%), vasoplegic shock in 549 (28%) and dysoxic shock in 219 (12%) patients. Mean age was 60 years, 47% were male and the majority was admitted form the emergency department (47%). The lung was the principal source of infection, followed by the urinary tract and abdominal. Overall, the four groups presented significant differences in APACHE II and SOFA scores (*P *< 0.001 for both), dysoxic shock being the most severe group. In *post-hoc *analysis, patients in the severe sepsis group presented similar SOFA score to patients in the cryptic shock group (*P *= 0.20). Overall, 28-day crude survival was different between groups (*P *< 0.001), being higher for the severe sepsis group (69%, *P *< 0.001 vs. other), similar between cryptic and vasoplegic shock (53%, *P *= 0.39) and lower for dysoxic shock (38%, *P *< 0.001 vs. other). In an adjusted analysis considering age, APACHE II and SOFA, the 28-day survival remained different between groups (*P *< 0.001) and the hazard ratio for the dysoxic shock group was the highest: HR 2.99 (95% CI 2.21 to 4.05).

## Conclusions

Current definitions for severe sepsis and septic shock include four different phenotypes, which should be considered for epidemiology purposes, customizing treatment goals and inclusion criteria for future studies. Although previous studies showed similar outcomes between cryptic shock and overt septic shock (vasoplegic and dysoxic profile), we demonstrated that cryptic shock is similar only to vasoplegic shock.
